# 

**Published:** 1867-11

**Authors:** 


### CONTENTS.

### ORIGINAL CONTRIBUTIONS.

ART. XLVII. How far do the Facts accompanying the Prevalence of Epidemic Cholera in Chicago, during the Summer and Autumn of 1866, Throw light on the Etiology of that Disease. By N. S. Davis, M.D., Professor of Principles and Practice of Medicine and of Clinical Medicine, Chicago, Ill., ........ 64[illegible]

ART. XLVIII. Tracings of the Pulse with Marey's Sphygmograph. By H. A. Johnson, M.D., Prof. Diseases of the Circulatory and Respiratory Organs, in Chicago Medical College, ........ 659

### FOREIGN CORRESPONDENCE.

The International Medical Congress, ........ 662, 665

### CORRESPONDENCE.

Laryngoscopy. By H. A. Johnson, M.D., ........ 672

### THE CLINIQUE.

Bowel-Affections of Children. By Prof. N. S. Davis, ........ 672

### PROCEEDINGS OF SOCIETIES.

Morgan County Medical Society, ........ 681 684

### SELECTIONS.

Locomotor Ataxia, ........ 687

### BOOK NOTICES.

The Physiology of Man. By Austin Flint, Jr., M.D., ........ 691

Medical uses of Electricity. By G. M. Beard, M.D., & A. D. Rockwell, M.D. 692

Is It I? By Horatio Robinson Storer, M.D., ........ 692

Transactions of the American Medical Association, Vol. XVIII, ........ 693

### EDITORIAL.

Prostitution and Syphilis, ........ 693

International Congress, ........ 694

The Lecture Season, ........ 694

American Journal of the Medical Sciences, ........ 695

The Medical Gazette, ........ 695

L'Evenement Medical, ........ 695

A New Process for Preparing Anatomical Specimens, ........ 696

Liebig's Artificial Milk, ........ 696

Heat as a Resuscitating Agent, ........ 697

Citrate of Soda in Glucosuria, ........ 697

Phosphorus Pills, ........ 697

Death from Swallowing Two Ounces of Chloroform, ........ 697

Mortality Report, ........ 699

Density of Ozone, ........ 700

Money Receipts, ........ 701

To Physicians, ........ 701

